# Facile Fluorescence “Turn on” Sensing of Lead Ions in Water via Carbon Nanodots Immobilized in Spherical Polyelectrolyte Brushes

**DOI:** 10.3389/fchem.2018.00470

**Published:** 2018-10-09

**Authors:** Yuchuan Tian, Antonios Kelarakis, Li Li, Fang Zhao, Yunwei Wang, Weihua Wang, Qingsong Yang, Zhishuang Ye, Xuhong Guo

**Affiliations:** ^1^State-Key Laboratory of Chemical Engineering, East China University of Science and Technology, Shanghai, China; ^2^School of Physical Sciences and Computing, University of Central Lancashire, Preston, United Kingdom; ^3^The Alle Chemical Company, Shandong, China; ^4^Engineering Research Center of Materials Chemical Engineering of Xinjiang Bingtuan, Shihezi University, Shihezi, China

**Keywords:** spherical polyelectrolyte brushes, SAXS, carbon dots, fluorescence sensor, lead ions

## Abstract

Heavy metal detection has become very important for the protection of water resource. In this work, a novel controllable probe is presented for the sensitive detection of Pb^2+^ in aqueous solutions. The probe was synthesized via the immobilization of surface functionalized carbon dots (named as CAEA-Hs) into the shell of the spherical polyelectrolyte brushes (SPB). The fluorescence of CAEA-H was firstly “turned off” via electrostatic interaction induced quenching. Based on the aggregation induced emission enhancement (AIEE), the fluorescence of the immobilized CAEA-H could be specifically turned on via the aggregation of the SPB particles. This fluorescence “turn on” sensor could selectively detect Pb^2+^ among five different metal ions with a relatively wide detecting range (0–1.67 mM) and good linear relationship (*R*^2^ = 0.9958). Moreover, the aggregating behavior and nano-structure of CAEA-H loaded SPB have been systematically analyzed via small angle X-ray scattering, turbidity titration, and Zeta-potential measurement. Based on a series of control experiments, we finally gain an insight into the sensing mechanism of this novel sensing probe. This contributed a proof of concept demonstration that sensitive and selective chemical detection can be achieved via a C-dot/SPB synergistic platform.

## Introduction

In recent years, carbon nanodots (C-dots) has become one of the most fascinating research topics in both fluorescent (Zuo et al., 2016) and electrochemical fields (Qi et al., 2016). C-dots are nano-carbon materials composed of carbogenic cores with functional groups (hydroxyl, carboxyl, amino etc.) (Krysmann et al., 2011). Particularly, because of their excellent fluorescence ability such as high quantum yields (Krysmann et al., 2011) and low toxicity (Tao et al., 2012) in biologic environment, C-dots stand out from the traditional fluorescent quantum dots and are gradually being regarded as the potential benign alternatives to heavy-metal-based quantum dots. As a result, researchers have explored various applications of C-dots in bio-imaging (Yang et al., 2009), cancer therapy (Huang et al., 2012; Hola et al., 2014), photocatalysis (Li et al., 2010), and chemical sensing (Wang and Hu, 2014). Among them, using C-dots as chemical sensors has drawn more and more attention due to their large specific surface area, good electrical conductivity, and high fluorescent emission (Fernandes et al., 2015). Depending on different sensing mechanisms, C-dots can be exploited in testing various analytes such as heavy metal ions (Guo et al., 2013, 2015), proteins (Freire et al., 2018), nitrocompounds (Campos et al., 2016), and other bioanalytes (Niu and Gao, 2014).

Heavy metal ions are causing growing environmental problems and can do severe damage to the well-being of mankind. Thus finding an efficient way to detect heavy metal ions in aqueous solution has become imperative. To date, various C-dots induced heavy metal sensors have been reported (Liu et al., 2011; Wee et al., 2013; Li et al., 2018; Luo et al., 2018; Tabaraki and Sadeghinejad, 2018). In principle, two distinct mechanisms are involved for the heavy metal detection by C-dots: fluorescence quenching (Sharma et al., 2017) and electrochemical redox reaction (Li et al., 2018). By modifying electrodes with different C-dot hybrids, researchers can prepare electrochemical sensors with low detection limit in a wide linear range. However, such sensing methods still have certain drawbacks, such as complicated electrode-modifying process, the use of sophisticated instruments and rather time-consuming sample preparation process. Consequently, there is a growing trend of using C-dot-based fluorescent sensor to detect heavy metal ions.

In order to achieve high selectivity and sensitivity, researchers tend to employ different precusors as carbon source and nitrogen source (Liu et al., 2011; Wee et al., 2013; Luo et al., 2018), or modify the surface of C-dots with different functional groups (Shen et al., 2013). However, in all these methods, the selectivity and sensitivity of fluorescent sensors are largely depended on C-dots themselves and the interaction between C-dots and the analytes, resulting in inevitable limitations. Based on that, fluorescence “turn on” sensors have been developed in recent years. In most cases, they are achieved by combining C-dots with organic or inorganic quenchers at first as “off-state,” followed by interaction with the analyte to reach the “on-state.” Yuan et al. (2014) developed a “turn on” sensing platform by modifying C-dots with bis-(dithiocarbamato) copper (II) (CuDTC_2_-CDs). The fluorescence of C-dots could be quenched by CuDTC_2_-CDs through electron and energy transfer process. Further addition of Hg^2+^ could significantly turn on the fluorescence of CuDTC_2_-CDs by displacing Cu^2+^ in the complex with Hg^2+^. Compared with “turn off” sensor based on fluorescence quenching mechanism, “turn on” fluorescence sensors are more desirable due to the high selectivity and wider detection range (Wei et al., 2012). Recently, based on aggregation induced emission enhancement (AIEE), a novel class of fluorescence “turn on” sensors has been proposed (Liu et al., 2016; Wang et al., 2016; Xu et al., 2018). Xu et al. (2018) prepared a fluorescent probe based on AIEE to selectively and sensitively detect Hg^2+^. C-dots were co-doped with nitrogen and sulfur (N, S-CDs). The resulting C-dots were well-dispersed in water and had a “turn-on” fluorescence response to Hg^2+^.

In order to optimize the AIEE based “turn on” sensing, it is important to understand the mechanism of the relevant fluorescence enhancing phenomenon. In the previous studies (Wang et al., 2016; Xu et al., 2018), several characterization methods were chosen to study the mechanism of AIEE: fluorescence emission spectroscopy, X-ray photoelectron spectroscopy (XPS), UV-vis spectroscopy, and transmission electron microscope (TEM). However, none of them, except for TEM, could directly confirm the behavior of aggregation of the bonded C-dots complex. Even in TEM characterization, during the sample preparation process, the nanoparticles will aggregate due to the evaporation of the solvent. Therefore, other more explicit characterization methods are needed to better examine the mechanism of AIEE.

Small angle X-ray scattering (SAXS) has proven to be one of the most efficient ways to understand the structure of nanoparticles (Wang et al., 2014, 2015; Tangso et al., 2015). For complex systems, SAXS is especially of great advantage since it is capable to analyze the nano-composites *in-situ* within various environments and give us a detailed information about the inner structure (Tian et al., 2016), load distribution (Wang et al., 2014), nano-shape (Polte et al., 2010), and even the aggregation behavior (Mathew et al., 2004). Therefore, we envision that SAXS could be a powerful tool to understand the AIEE process during the fluorescent “turn on” process.

Due to the strong electrostatic force among the polyelectrolyte chains and the controllable behavior change, the core-shell shaped spherical polyelectrolyte brushes (SPB) have been known as the ideal candidate for immobilizing functional nanoparticles such as proteins (Wang et al., 2013), metals (Sharma and Ballauff, 2004; Mei et al., 2007), and quantum dots (Liu et al., 2014).

For most of the C-dots involved fluorescent metal sensors, the sensing mechanism is based on the direct fluorescence quenching between sensors and Metal ions (Liu et al., 2011; Wee et al., 2013; Luo et al., 2018). To the best of our knowledge, only two articles (Wang et al., 2016; Xu et al., 2018) studied the detection of heavy metals such as Hg^2+^, or Fe^3+^ that is based on the AIEE process using C-dots as templates for the AIEE detection of Pb^2+^. Moreover, in our study, SAXS was employed for the first time to prove the specific aggregation behavior between C-dots-SPBs nanoparticles under the presence of Pb^2+^.

**Scheme 1:**
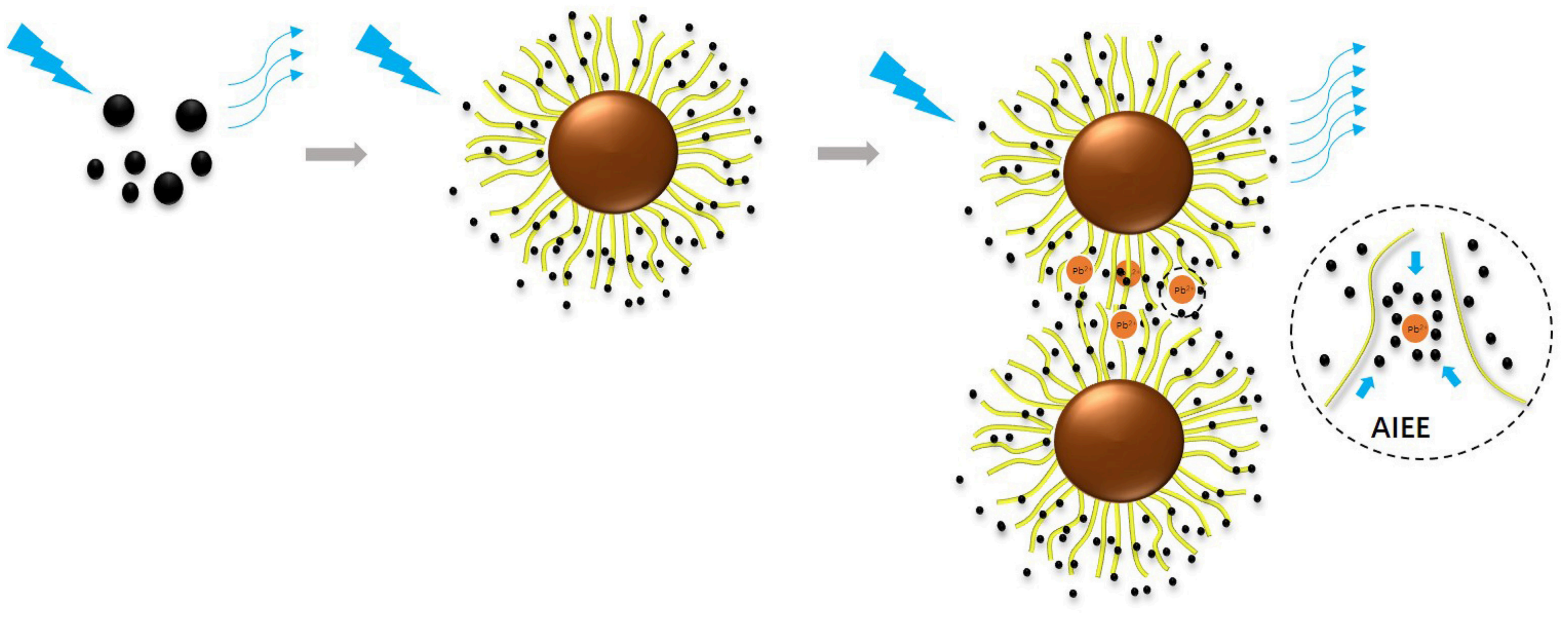
Fluorescence turn on sensing of Pb^2+^ via immobilization C-dots into SPB.

Herein, we design a novel Pb^2+^ fluorescence “turn on” sensing probe by immobilizing C-dots into the brush shell of SPB in water solution. Based on previous study, excitation-dependent C-dots were synthesized with citric acid as carbon-source, and ethanolamine as N-source (CAEA). The surface of C-dots was further functionalized with extra carboxyl and hydroxyl groups via reaction with nitric acid (named as CAEA-H). Using core-shell poly(styrine)-poly(acrylic acid) SPB (named as PAA brushes) as carrier, we successfully immobilized the obtained C-dots into the brush layer of PAA chains (named as CAEA-H-PAA brushes). Due to electrostatic quenching, the emission fluorescence of the CAEA-H was significantly “turned off” after the C-dots were immobilized in SPB. By fluorescence titration for different metal ions in water solution, we obtained a selective and sensitive detection of Pb^2+^ via CAEA-H-PAA brushes nanocomposite sensor with a wide linear detecting range. Specifically, we found that among several different metal ions (including Ag^+^, Pb^2+^, Zn^2+^, Al^3+^, and Cd^2+^), only Pb^2+^ could efficiently “turn on” the quenched emission fluorescence through the AIEE process between the nanocomposite sensor and Pb^2+^. Moreover, the mechanism of the AIEE process was systematically studied by fluorescence emission spectra, SAXS, Zeta-potential measurement, and turbidity titration (As shown in Scheme 1).

## Experimental section

### Materials

Citric acid (CA), nitric acid, and potassium chloride were purchased from Sigma-Aldrich and ethanolamine (EA) was purchased from Alfa Aesar. Acrylic acid (AA), K_2_S_2_O_8_ (KPS), styrene, sodium chloride (NaCl) and sodium dodecyl sulfonate (SDS) were purchased from Shanghai Reagent Company. 2-Hydroxy-4′-hydroxyethoxy-2-methyl propiophenone (HMP), and methacryloyl chloride were purchased from Tokyo Chemical industry Co. Silver nitrate (AgNO_3_), lead chloride (PbCl_2_), zinc chloride (ZnCl_2_), and aluminum chloride (AlCl_3_) were purchased from Shanghai Reagent Company. Cadmium chloride was purchased from Titan Reagent Company. Sodium dihydrogen phosphate (NaH_2_PO_4_) and iron(III) chloride (FeCl_3_) was obtained from Macklin Reagen. Manganese(II) chloride (MnCl_2_), potassium chloride (KCl), calcium chloride anhydrous (CaCl_2_), and disodium hydrogen phosphate (Na_2_HPO_4_) was purchased from Shanghai Lingfeng Reagent. Nickel(II) Chloride anhydrous (NiCl_2_) was from Damas Beta reagent company, cobaltous(II) chloride anhydrous (CoCl_2_) and Barium chloride anhydrous (BaCl_2_) was from Infinity Science. Photo-initiator 2-[p-(2-hydroxy-2-methyl propiopenone)]-ethyleneglycol methacrylate (HEMEM) was synthesized in our laboratory according to our previous paper (Guo et al., 1999). Ultrapure water was purified by reverse osmosis (Millipore Milli-Q) and used in all experiments. Styrene and AA were used after reduced pressure distillation and stored in a refrigerator at 4°C. KPS was recrystallized in water. All the other materials were used without further purification.

### Synthesis and modification of C-Dots

The synthesis of C-dots was based on the previous publication (Krysmann et al., 2011). In brief, C-dots were synthesized by controlled pyrolysis of CA and EA (molar ratio 3:1) at 180°C for 30 min under reflux, followed by a 30 min further heating at 230°C in the absence of the reflux condenser. Then the samples were pyrolyzed in the oven at 300°C for 1 h. Afterwards the product (named CAEA) was treated with 3 M nitric acid with a volume ratio 1:3 at 120°C for 16 h, in order to modify the C-dot surface with extra carboxyl and hydroxyl groups (CAEA-H). In both cases, the C-dot solution was dialyzed against ultrapure water through SnakeSkin Pleated Dialysis Tubing membrane (with a molecular weight cutoff of 3,500 Da) until the conductivity of the outer solution became constant.

### Synthesis of PS-PAA spherical polyelectrolyte brushes

The PS core was synthesized using a conventional emulsion polymerization according to the literature (Guo et al., 1999). In short, 0.74 g KPS, 0.24 g SDS, and 10 g styrene were added to the flask with 150 mL water, followed by the repeated degassing and subsequent flushing with nitrogen for at least 3 times. The reaction was carried out at 80°C for 2 h under the nitrogen atmosphere with a stirring rate of 300 rpm. Then 1 g photo-initiator HMEM dissolved in 7 g acetone was slowly added to the system under starved condition (0.05 ml/min). After another reaction for 2.5 h, the obtained PS core was purified in Milli-Q water by dialysis for 3 days.

The PAA brushes were synthesized through photo-emulsion polymerization. In a typical run, 100 g PS core solution was added to a 500 ml three-necked flat-bottomed quartz photo-reactor. The amount of AA added in mole is equal to that of styrene in PS core. Then more water was added to the reactor until the mass of the whole reaction system reached 400 g. After the addition of AA to the PS core latex, the system was degassed by repeated evacuation and flushing of nitrogen for at least three times. Then photo-emulsion polymerization was accomplished after UV radiation at room temperature with vigorous stirring for 2.5 h. The obtained SPB (with PAA brushes) were purified by ultra-filtration until the conductivity of outer water became constant.

### Immobilization of C-dots into SPB (CAEA-H-PAA brushes)

In our study, we chose the carboxyl-group-rich CAEA-H as the template for the fluorescence “turn on” sensor. By adding C-dots solution into SPB solution dropwise under magnetic stirring for 30 min, CAEA-H immobilized in PAA brushes (CAEA-PAA brushes) with different mass ratios were prepared. The concentration of CAEA-H was fixed at 0.3 mg/mL, while two concentrations of PAA brushes were used: 1.2 and 3.6 mg/mL, respectively. Additionally, the same amount of CAEA was immobilized onto PAA brushes in the same manner as a control group.

### Fluorescence “turn on” sensing of metal ions

We chose 5 different metal salts (AgNO_3_, PbCl_2_, ZnCl_2_, AlCl_3_, and CdCl_2_) to test the sensing ability of the CAEA-H-PAA brushes. 0.6 mL CAEA-H-PAA solution was despersed into 2.4 mL phosphate buffer solution (PBS) (pH 7.4, 10 mM). Two milliliters of the obtained solution was put into a 3 mL quartz cuvette. Then different volumes (from 20 to 100 μL) of 5 metal ion solutions (concentration = 0.05 M) were added into the cuvette respectively, followed by the monitor of fluorescence emission spectrometer (F97pro, Shanghai Lengguang Technology Fluorometer). The fluorescence quenching data was collected in the form of *I*/*I*_0_, where *I*_0_ and *I* are the fluorescence emission before and after adding metal ions. Also, a control experiment was carried out by studying the unmodified CAEA-PAA brushes in the same manner.

### Structure characterization by small angle X-ray scattering

In the SAXS test, 0.1 mL of sample solution was placed in the sample cell wrapped by polyimide films on both sides. The scattering intensity data of C-dots solution, PAA solution and CAEA-H-PAA-metal ions complex solution were collected by beamline BL16B1 at Shanghai Synchrotron Radiation Facility (SSRF) respectively. The concentration of CAEA-H was fixed at 0.3 mg/mL at 10 mM pH 7.4 PBS buffer, and the concentration of metal ions changed from 0 to 1.67 mM with an increment of 0.33 mM. The testing distance between samples to the detector was 2 or 5 m.

### Other characterizations

The size and size distribution of C-dots and CAEA-H-PAA brushes were studied by transmission electron microscopy (TEM, JEOL JEM-2010) at 200 kV. The zeta potential and the diameter of the SPB was collected via dynamic light scattering (DLS, Particle Sizer NICOMP 380 ZLS instrument). Turbidity (%T), reported as 100 – %T, was obtained with a Brinkmann PC 950 colorimeter (420 nm filter). FTIR spectra were recorded using a Nicolet IR2000 spectrophotometer at room temperature. The agglomerates and fluorescence of C-dots-SPB was observed via a polarized light microscope (2500P, LEIKADAM) with a fluorescence module (excitation wavelength λ = 390–410 nm).

Normally, the experiments were repeated 3 times to achieve a satisfying reproducibility.

### SAXS theory

#### Analysis of SPB

Generally, the contribution of the scattering intensity of a single SPB consists of the following 3 parts: (Rosenfeldt et al., 2004)

(1) $$\begin{array}{r} I_{1}\left(q\right)=I_{fluct}\left(q\right)+I_{ps}\left(q\right)+I_{cs}\left(q\right) \end{array}$$

Here *q* means the scattering vector, and *I*_*cs*_(*q*) denotes the scattering intensity that results from the core-shell structure of SPB. Furthermore, *I*_*cs*_(*q*) is governed by the radial excess electron density distribution of the polyelectrolyte chains: Δρ^*e*^(*r*) = ρ^*e*^(*r*) – ρ^*e*^*M*, where ρ^*e*^(*r*) is the electron density distribution profile of SPB, and ρ^*e*^*M* is the electron density distribution of the background. *I*_*cs*_(*q*) can be calculated from *B*^2^(*q*) where *B*(*q*) is the scattering amplitude: (Dingenouts et al., 1998)

(2) $$I_{cs}(q)=B^{2}(q)={\left[4\pi \int_{0}^{R}\Delta \rho^{e}(r)\frac{\sin(qr)}{qr}r^{2}dr\right]}^{2}$$

Moreover, for polydisperse system, the *I*_*cs*_(*q)* can be easily obtained from the sum of the *B*${}_{i}^{2}$(*q*) weighted by the number density (De Robillard et al., 2000).

The second part *I*_*fluct*_ is mainly attributed to the fluctuations from the polyelectrolyte chains. It can be simplified with the Lorentzian equation (De Robillard et al., 2000):

(3) $$\begin{array}{r} I_{fluct}\left(q\right)=\frac{I_{fluct}\left(0\right)}{1+\xi^{2}q^{2}} \end{array}$$

where ξ is the spatial extension of the fluctuation. Both *I*_*fluct*_(0) and ξ are adjustable parameters in this case.(Rosenfeldt et al., 2004) At higher *q* values, *I*_*fluct*_(*q*) plays a predominant role in the overall scattering intensity.

The third part *I*_*ps*_(*q*) comes from the contribution of the PS core, which is negligible in our case with a rather narrow *q* distribution (Rosenfeldt et al., 2004).

#### Analysis of C-Dots

Similar to PS core, a single carbon dot can be modeled as a “hard” sphere with a uniform electron density distribution inside, Δ$\rho_{CDs}^{e}$ (541.8 e^−^/nm^3^) and a narrow shell with a rather lower density, Δ$\rho_{s}^{e}$. This means *r*_*core*_ and Δρ_*CDs*_ are much higher than *d*_*shell*_ and Δ$\rho_{s}^{e}$, respectively. Hence, it is reasonable to neglect the contribution of shell, and the scattering intensity of C-dots *I*_*CDs*_ could be calculated as follow:

(4) $$I_{CDs}(q)=B^{2}(q)={\left[4\pi \int_{0}^{R}\Delta \rho^{e}{}_{CDs}\frac{\sin(qr)}{qr}r^{2}dr\right]}^{2}$$

#### Analysis of C-Dots-SPB

In theory, for a binary system composed of a big sphere and several small spheres (Rosenfeldt et al., 2004), the overall scattering intensity is the square of the sum of the two scattering amplitude *B*(*q*) of different spheres. Based on the previous studies (Rosenfeldt et al., 2004; Wang et al., 2017), at small scattering angles, for monodisperse system, the analysis process can be further simplified as follows: the immobilized small particles are deemed to increase the electron density of the large sphere around its surface, resulting in the increase of *I*_*cs*_ at smaller q region (*q* < 0.4 nm^−1^). Whereas, in a higher q region (*q* > 0.6 nm^−1^), the scattering intensity is the sum of *I*_*fluct*_ and the intensity of the free small spheres having the same concentration to the C-dots-SPB solution [*I*_*CDs*_(*q*)]. Thus, for our system, at a rather small q region (0.05–1.5 nm^−1^), the scattering intensity of C-dots-SPB could be obtained as follows:

(5) $$\begin{array}{c} I_{1}(q)=I_{fluct}(q)+I_{CDs}(q)+I_{cs}(q) \end{array}$$

#### Fitting model

**Figure 1:**
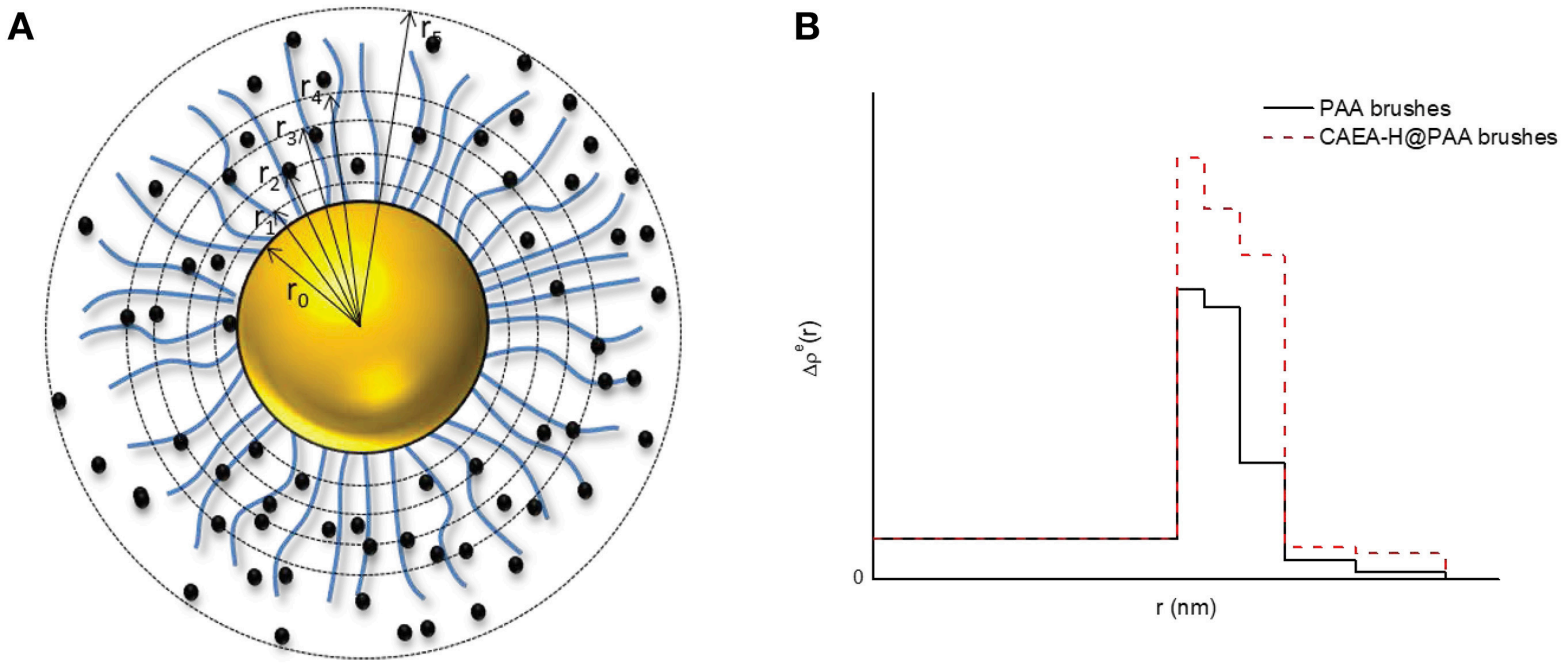
The five-layer model for the distribution of Δρ^e^ for C-dots- SPB. **(A)** Schematic illustration of C-dots-SPB structure; **(B)** Δρ^e^(*r*) of PAA brushes and C-dots-PAA brushes.

The scattering intensity can be calculated from Equation (2). Hence the critical part is to find the ideal fitting model for the system. The 5-layer model has proven to be an efficient model to simplify the fitting process without scarifying accuracy (Dingenouts et al., 1998; De Robillard et al., 2000; Rosenfeldt et al., 2004; Wang et al., 2017). As shown in Figure 1, the distribution of the shell is divided into 5 layers where in each layer, the Δρ^*e*^(*r*) is a constant.

Hence, the integral of Equation (2) could be calculated as follows:

(6) $$\begin{array}{l} I_{cs}=B_{i}{\left(q\right)}^{2}\\ \;=\{\begin{array}{l} {\left[4\pi \Delta \rho_{0}{}^{e}\left(\frac{\sin qr_{0}-qr_{0}\cos qr_{0}}{q^{3}}\right)\right]}^{2}\left(i=0\right)\\ {\left[4\pi \sum_{j=i-1}^{i}\Delta \rho_{j}{}^{e}\left(\frac{\sin qr_{j}-qr_{j}\cos qr_{j}}{q^{3}}\right)\right]}^{2}\left(1\leq i\leq 5\right) \end{array}\; \end{array}$$

Δρ${}_{0}^{\text{e}}$ is the excess electron density of PS core (6.4 e/nm^3^). Δρ^e^_*j*_ is the given excess electron density of each layer, and *r*_*j*_ is the radius of each layer. When C-dots are loaded into brush shell, we can accordingly increase the Δρ^e^_*j*_ of each layer to fit the scattering intensity increase at small *q*-values (< 0.4 nm^−1^, as shown in Figure 1B).

For polydisperse system with a narrow size distribution, Gaussian distribution *G*(*r*) is used to model the dispersity of both C-dots and C-dots-SPB in solution: (De Robillard et al., 2000; Rosenfeldt et al., 2004).

(7) $$G(R,R_{0},\sigma )=\frac{1}{\sigma \sqrt{2\pi }}\exp(-\frac{{\left(R-R_{0}\right)}^{2}}{2\sigma^{2}}),\int_{0}^{\infty }G(r)d(r)=1$$

where R_0_ is the average radius, and δ is the standard deviation. Therefore, the intensity could be calculated from Equation (8):

(8) $$\begin{array}{l} I(q)=4\pi \gamma \frac{N}{V}S(q)I_{cs}(q)=4\pi \gamma \frac{N}{V}\\ \;S(q)(\int_{0}^{\infty }\int_{0}^{\infty }G(r_{c})G(r_{s})(\sum_{i=0}^{5}B_{i}{\left(q\right)}^{2})d(r_{c})d(r_{s})) \end{array}$$

where γ is the adjustable parameter, N/V is the number density of the nanoparticles, and *S*(*q*) is the structure factor among particles that only influences the intensity at small *q*-values. For dilute system with noninteracting particles, *S*(*q*) can be simplified to 1.

However, it is worth noting that, for systems with a broad size distribution or nonnegligible particle interactions, it is no longer acceptable to use Gaussian distribution to build the distribution model (Wang et al., 2017). Obviously, for an original system that fits Gaussian distribution, the further special binding-up between several particles would inevitably cause the inconsistency to the Gaussian distribution. Moreover, when aggregation happens, the structure factor S(q) should be also taken into account (Hilfiker et al., 1990; Wang et al., 2017).

Hence, for our further discussion concerning the aggregation of SPB, the fitting model with Gaussian distribution can no longer serve its purpose. Moreover, the corresponding aggregation behavior with the addition of Pb^2+^ could be directly confirmed from the raw scattering curves based on the elevation of the first wave hollow (Hilfiker et al., 1990; Wang et al., 2017). As a result, a detailed observation of the scattering intensity is reliable enough to confirm the aggregation behavior of C-dots-SPB system without further fitting.

## Results and discussion

### Characterizations of CAEA-H and CAEA-H-PAA brushes

**Figure 2:**
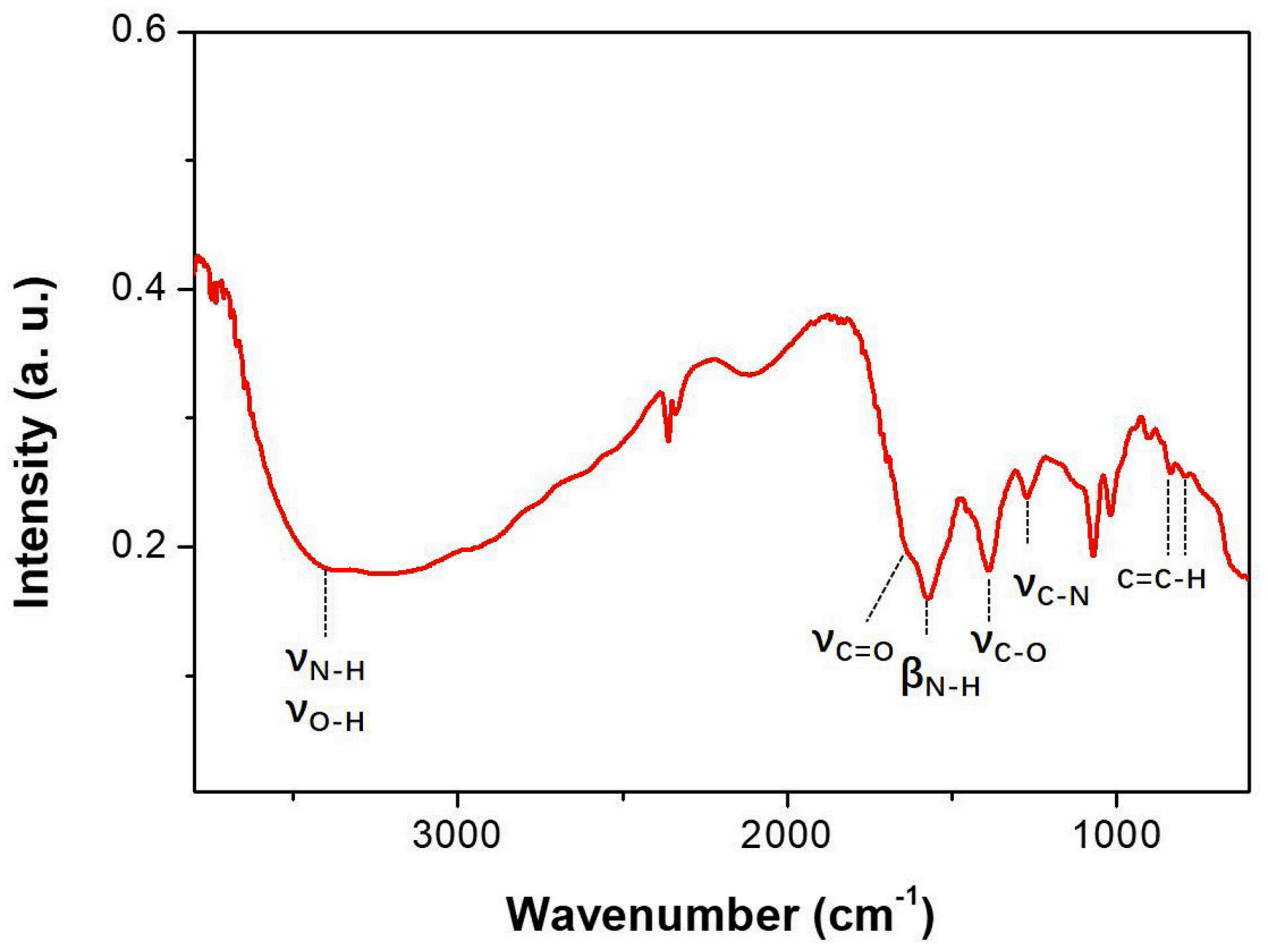
FTIR spectra of CAEA-H.

The FTIR results of CAEA-H are shown in Figure 2. The broad adsorption peak from 2,500 to 3,650 cm^−1^ along with the adsorption around 1,000 cm^−1^ could be attributed to the vibration of -COOH, N-H and O-H. (Shen et al., 2013; Luo et al., 2018). And the peaks at 1,640 and 1,563 cm^−1^ are the vibration of C = O and C-N, respectively (Krysmann et al., 2011). The peak at 1,382 cm^−1^ represents the adsorption bands arising from -OH groups (Freire et al., 2018). The peak at 1,265 cm^−1^ denotes the vibration of C-N (Luo et al., 2018). And the peaks at 831 and 792 cm^−1^ are attributed to the bending vibration of the C = C-H bond from the sp^2^ carbon (Shen et al., 2013). The FTIR adsorption of CAEA-H indicates the presence of different functional groups such as -COOH, -NH_2_, and -OH at the C-dot surface, as well as the carbogenic core with sp^2^ orbital (Krysmann et al., 2011).

**Figure 3:**
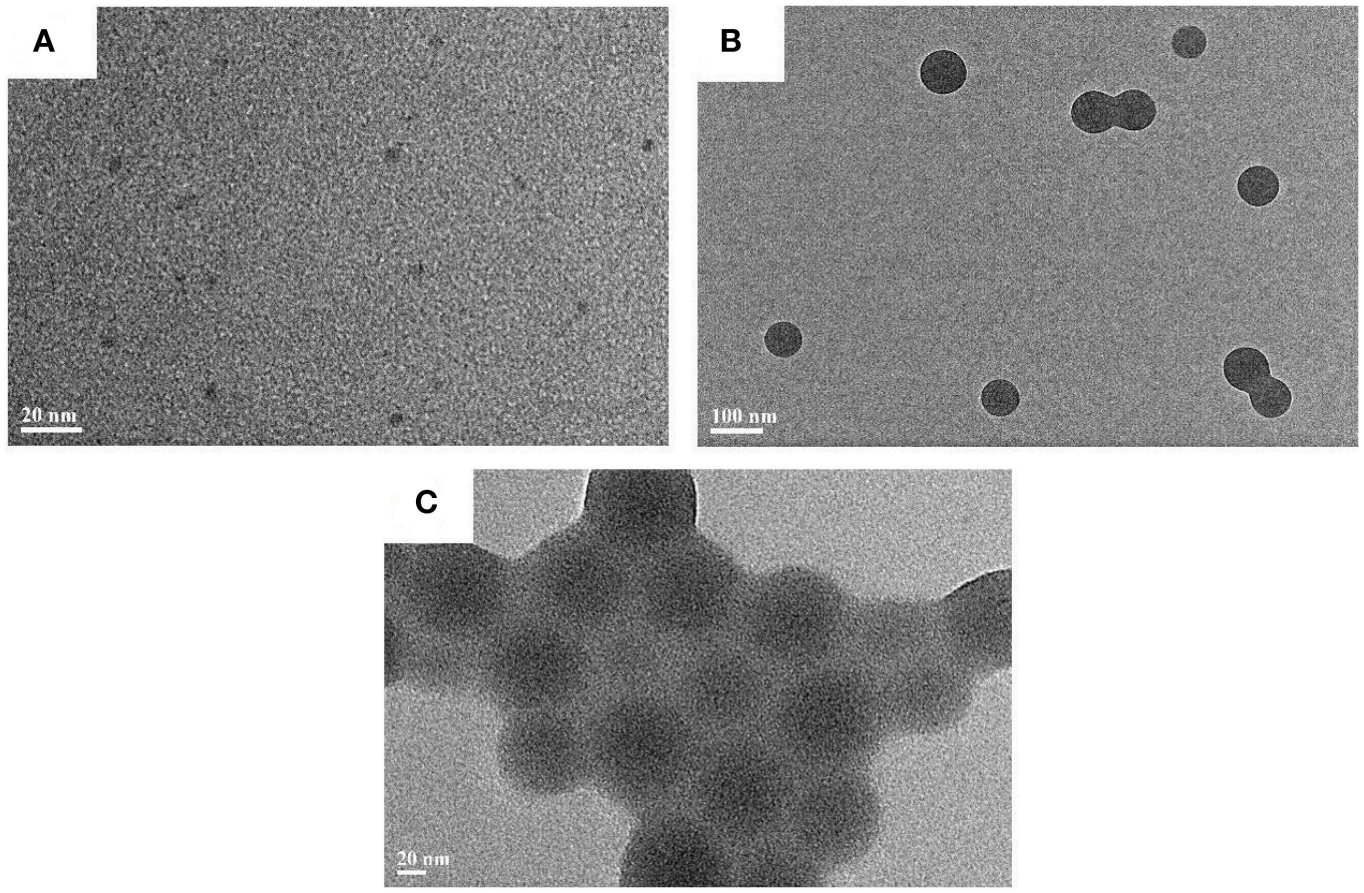
TEM results of C-dots and C-dots loaded particles: **(A)** CAEA-H, **(B)** PAA brushes, and **(C)** CAEA-H-PAA.

The morphology of CAEA-H and CAEA-H-PAA brushes were studied via TEM. As shown in Figure 3A, the CAEA-H particles were almost monodispersed with an average diameter 4.3 nm. This agrees well with the previous publications (Cao et al., 2007; Bourlinos et al., 2008; Ray et al., 2009). For PAA brushes, though a well-defined sphere structure can be observed from Figure 3B, the core-shell structure cannot be exhibited from the TEM image (Grünewald et al., 2015). As is shown in Figure 3C, it is hard to distinguish the CAEA-H from the surface of PAA brushes. That is probably due to the low contrast of the CAEA-H to the surrounding PAA brushes. Nevertheless, there were almost no unbonded C-dots found in the background, indicating that most C-dots were successfully immobilized inside PAA brushes.

**Figure 4:**
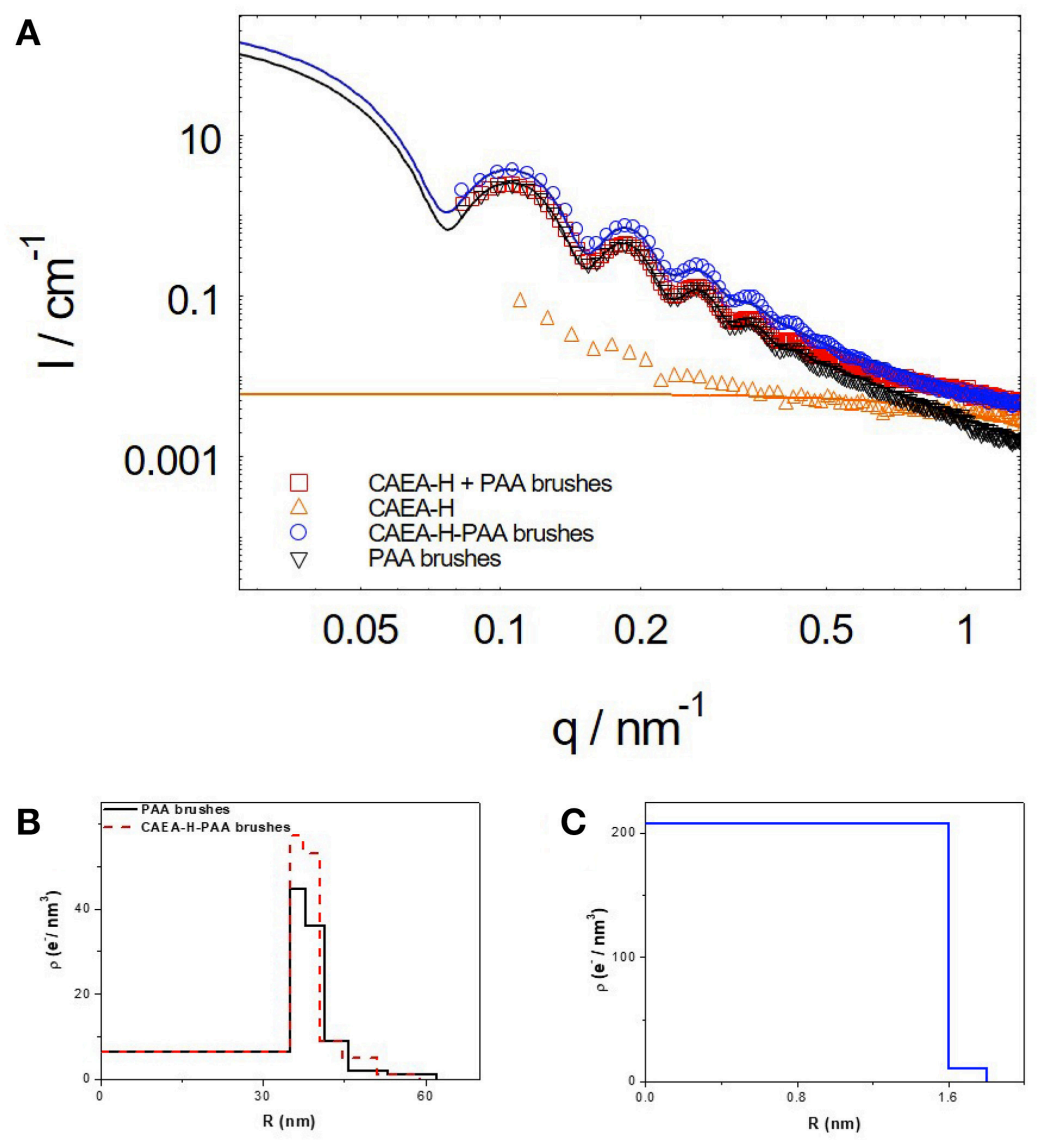
**(A)** SAXS results of CAEA-H, PAA brushes and C-dots-PAA brushes (the solid lines are the fitting curves). **(B)** Excess electron density distribution of CAEA-H-PAA brushes**. (C)** The excess electron density distribution of CAEA-H. The concentrations of CAEA-H and PAA brushes in SAXS tests were 0.3 and 3.6 mg/mL, respectively in an aqueous solution of 10 mM NaCl and pH 7.4.

SAXS can give us a better understanding of the nano-structure of CAEA-H-PAA brushes. As shown in Figure 4A, at small *q*-values (0.09–0.3 nm^−1^), the upturn of the scattering intensity of CAEA-H is caused by the partial aggregation of the CAEA-H (Wang et al., 2017). When CAEA-H was immobilized into PAA brushes, the *I*(q) of CAEA-H-PAA increased over a broad *q* range (0.083–1.5 nm^−1^) without any influence on the dispersity. Compared with the intensity that simply derived from the direct sum of *I*(q)_CAEA−H_ and *I*(q)_PAA_, we can discern a distinct elevation of the curve in the small q region (0.083–0.7 nm^−1^) for CAEA-H-PAA. This elevation mainly resulted from the addition of CAEA-H into the brush shell PAA. Whereas, at higher *q*-values (0.7–1.5 nm^−1^), the scattering intensity of (CAEA-H-PAA brushes) and (CAEA-H + PAA brushes) overlapped well each other. This suggests that the scattering intensity at higher *q* values of the mixed system is mainly governed by the combination of the intensity of free CAEA-H and the fluctuation from the polymer chains (Rosenfeldt et al., 2004).

Moreover, from the radial excess electron distribution profile (Figure 4B), we can obtain more specific details about the inner structure. The sharp increase of the electron density at the inner of the brush shell further proves the fact that most carbon dots are immobilized into the inner part of PAA brush shell. Due to the immobilization of C-dots, the electrostatic repulsions were weakened, resulting in the decrease of radius of the SPB from 62 to 59 nm, which is consistent with the DLS results (Wang et al., 2017) (Figure S1). The radius of CAEA-H, determined from its Δρ^*e*^(*r*) chart in Figure 4C, was 1.8 nm, which is slightly smaller than the TEM results (2.15 nm).

### Electrostatic interaction induced fluorescence quenching

It is generally believed that the fluorescence emission comes from the conjugated π state (C = C) from the core region, the functional groups around (C = O, -COOH, -OH, and -NH_2_), and the electronic state transitions (Krysmann et al., 2011; Zeng et al., 2017).

**Figure 5:**
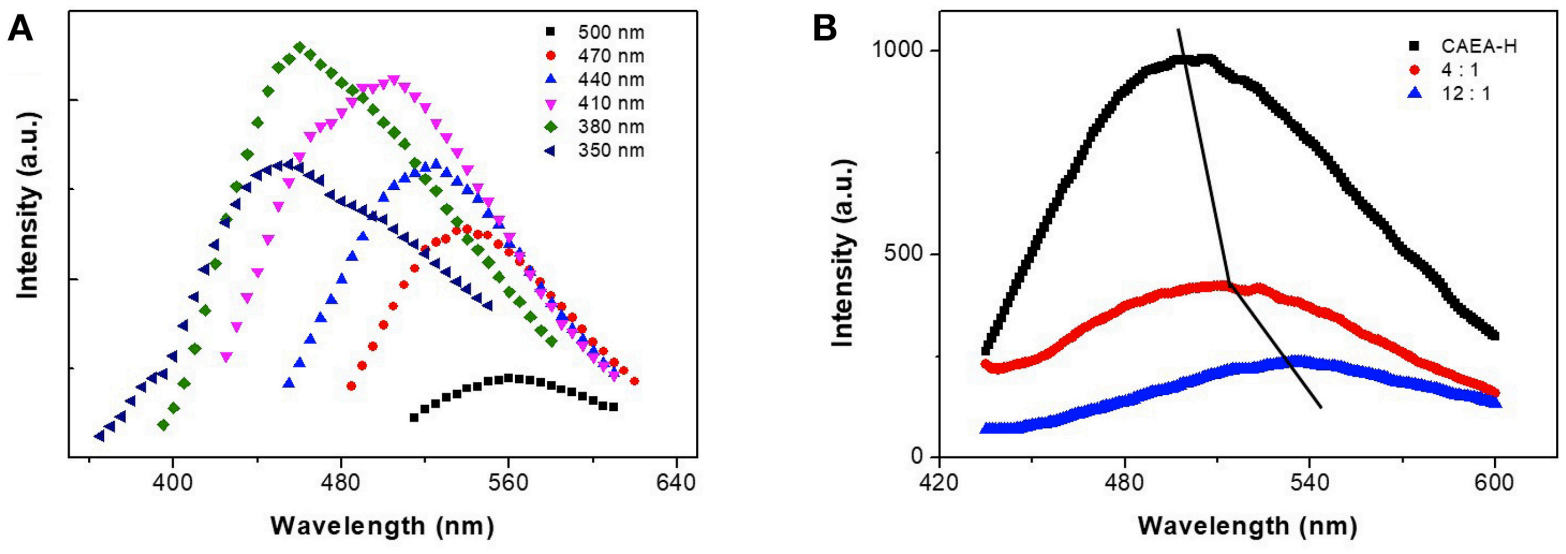
**(A)** Fluorescent emission spectra of CAEA-H at different excitation wavelength. **(B)** Emission spectra of CAEA-H-SPB with different mass ratios of SPB and C-dots at excitation wavelength 430 nm (the mass concentration of C-dots was fixed at 0.3 mg/mL).

As shown in Figure 5A, the fluorescent emission spectra of CAEA-H coincide well with our previous publication (Krysmann et al., 2011). A excitation wavelength dependent emission was observed for different excitation wavelength. The fluorescence emission increased as the increase of excitation wavelength. The excitation-dependent emission was attributed to the C = C in core region.

When comparing the emission spectra of CAEA-H-PAA brushes with different ratios of SPB to C-dots (0:1, 4:1, 12:1), we can clearly observe the “turn off” of emission after PAA brushes were added to the CAEA-H system at Figure 5B. Upon increasing the amount of the PAA brushes, the fluorescence quenching was more remarkable. In literatures (Itoh et al., 1984; Wang et al., 2004; Hu et al., 2005), it has been proved that the strong electrostatic force between polyelectrolytes and some counter ions could lead to strong enhancement of fluorescence quenching. Whereas in the context of our work, the electrostatic force between PAA chains (-COOH) and CAEA-H (-NH_2_) plays a major role in the fluorescence quenching between CAEA-H and PAA brushes. Besides that, there is a clear red-shift of the emission peaks in Figure 5B. It is possibly due to the polarity change caused by the immobilization of CAEA-H into PAA brushes (Hu et al., 2005). According to the fluorescence emission data in Figure 5B, the approximate immobilization amount of CAEA-H-PAA 1:12 obtained from the quenching efficiency (*I*/*I*_0_) was ca. 0.05 mg/mg-SPB. From SAXS results (Figure 4B), the amounts of C-dots could be approximately calculated from the difference between the excess electron distribution of SPB and CAEA-H-SPB. From the calculation the approximate immobilization amount was ca. 0.04 mg/mg-SPB. Within the error allowed, the immobilization amounts of C-dots for CAEA-H-PAA-1:12 could be 0.04 ± 0.02 (mg/mg SPB) (See Supplementary Material for the detailed calculation process).

**Figure 6:**
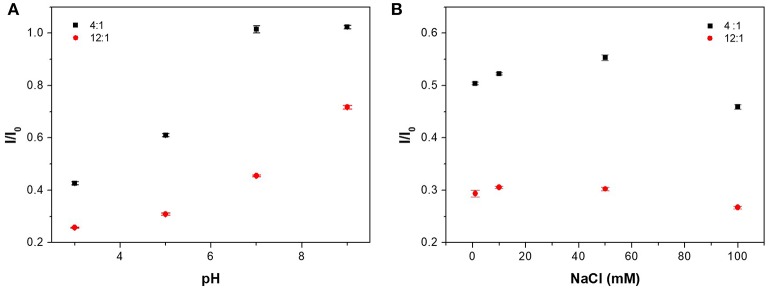
Influence of pH **(A)** and salt concentration **(B)** on the emission ratio of CAEA-H-PAA at different mass ratios of PAA brushes and CAEA-H. NaCl concentration was 10 mM in **(A)**, and pH was 3 in **(B)**. The concentration of C-dots is fixed at 0.3 mg/mL.

Here, we use the normalized emission ratio (I_CAEA−H−PAA_/I_CAEA−H_) to study the fluorescence emission at different pH and salt condition. The emission ratio was higher when more SPB was added to the system. As shown in Figure 6A, at pH = 3 or 5, the emission ratio of CAEA-H-PAA-4:1 was low at “off-state.” At pH = 7 or 9, however, the emission ratio for CAEA-PAA-4:1 drastically increased to nearly 1 as “on-state” and remained stable. Similar to the case of proteins (Wang et al., 2013), the electrostatic interaction between C-dots and SPB is a combination of electrostatic repulsion and attraction. When pH increases, the electrostatic repulsion between the carboxyl groups on the PAA chains and CAEA-H becomes stronger due to the enhancement of ionization of -COOH group, resulting in the weakening of the overall electrostatic attraction between PAA brushes and CAEA-H. As a result, more free CAEA-H will exist in the solution which leads to the enhancement of fluorescence. After pH = 7, the repulsion is already larger than attraction force among PAA chains and CAEA-H, thus further increase of pH would no longer increase the emission ratio.

As for ionic strength at Figure 6B, for different amount of PAA brushes, similar trend of emission quenching at different NaCl concentrations has been found. In the low NaCl concentration range (0–10 mM), the quenching efficiency increased firstly, and then reached a maximum around 10 mM. This behavior could be attributed to the migration of the CAEA-H from inner brush shell to the outer solution due to the ion exchange with the Na^+^. At even higher salt concentrations, the existence of many counter ions in the solution would be confined around PAA shell, which would prominently screen the fluorescence of CAEA-H via electrostatic quenching (Itoh et al., 1984). The above discussion confirms that the quenching of CAEA-H with the presence of PAA brushes is mainly induced by the electrostatic interactions among CAEA-H and PAA chains.

### Facile detecting pb2^+^ of CAEA-H-PAA brushes in aqueous solution

**Figure 7:**
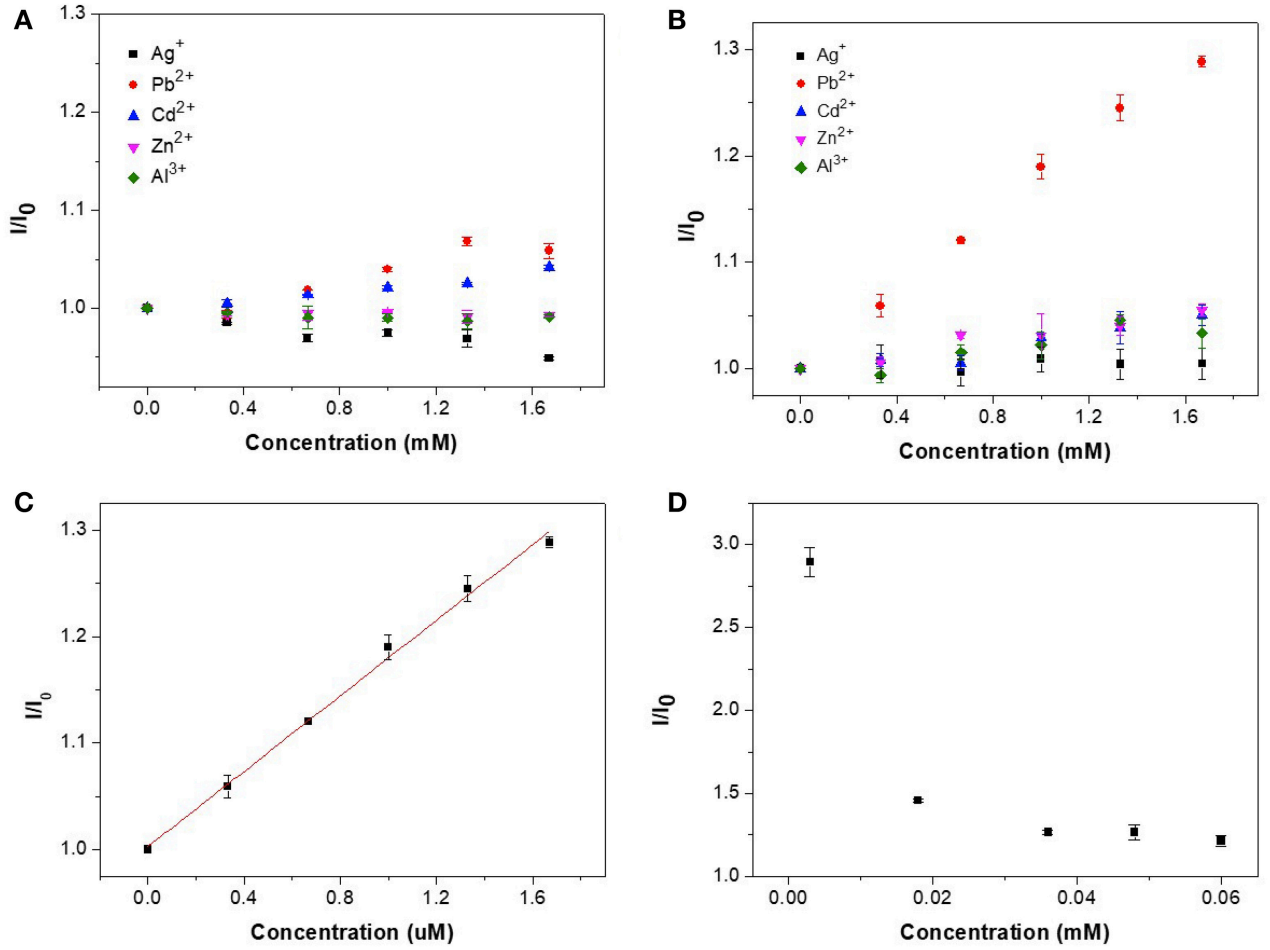
Fluorescence titration for different metal ions in **(A)** CAEA-H, and **(B)** CAEA-H-PAA (mass ratio of CAEA-H and SPB 1:12) aqueous solution. The concentration of CAEA-H was 0.06 mg/mL by dispersing samples into 10 mM PBS buffer (pH 7.4) in both solutions. **(C)** Linear fitting results of the I/I_0_ of CAEA-H-PAA SPB for detecting Pb^2+^. **(D)** Relationship between fluorescence emission and the concentration of CAEA-H-PAA brushes.

Figure 7 shows the detection of metal ions by CAEA-H and CAEA-H-PAA, where the mass ratio of CAEA and SPB is 1:12 and the samples are dispersed in 10 mM PBS buffer (pH 7.4). When there were no PAA brushes (Figure 7A), the increase in the concentration of different metal ions seems to have less impact on the fluorescence emission. Still, for heavy metals Pb^2+^ and Cd^2+^, a slight increase of the fluorescence emission (*I*/*I*_0_ ≈ 1.05) could be detected, this rather limited effect might be attributed to a change of C-dots electronic environment that results in stronger fluorescence. Moreover, a control experiment had been done via testing the emission ratio of the unmodified CAEA with Pb^2+^ (0.06 mg/mL CAEA at 10 mM PBS buffer with pH 7.4) (see Table S1). And it was found that there was no fluorescence enhancement with a emission ratio of 0.98. By contrast, the emission of CAEA-H in Figure 7A slightly increased under the same condition.

As for the SPB-imported CAEA-H-PAA brushes (Figure 7B), the nominalized emission *I*/*I*_0_ for detecting Pb^2+^ ions was remarkably improved from 1.05 to 1.28, whereas for other metal ions, the fluorescence only slightly increased with the increase of concentration. Moreover, a good linear relationship between the Pb^2+^ concentration and I/I_0_ was acquired. As shown in Figure 7C, the regression equation is I/I_0_ = 1.0028 + 0.1770 [Pb^2+^] with a *R*^2^ of 0.9958. The detecting limit was found to be 22.8 μM based on the calculation from 3δ/*s* (Wee et al., 2013), where δ is the standard deviation for 10 blank samples, and *s* is the slope of the calibration curve. The calibration curve for CAEA-H-PAA system exhibits an excellent linear relation with a considerably wide detecting range compared to other sensors using fluorescent methods (Liu et al., 2011; Wee et al., 2013; Li et al., 2018; Luo et al., 2018; Tabaraki and Sadeghinejad, 2018).

Due to the unique property for SPB to adsorb counter ions (Mei et al., 2007; Wang et al., 2013), the sensitivity of the sensor could be further increased by simply decreasing the sensor-to-target ratio (Wei et al., 2012). Experiment was carried out via decreasing the sensor concentration to 0.003 mM while the concentration of Pb^2+^ was fixed at 1.33 mM. As shown in Figure 7D, the sensitivity for detecting Pb^2+^ increased with the decrease of sensor concentration. This result extends the potential application of our CAEA-H-PAA brushes sensing platform with adjustable sensitivity.

### Selectivity of the CAEA-H-PAA for pb2^+^

**Figure 8:**
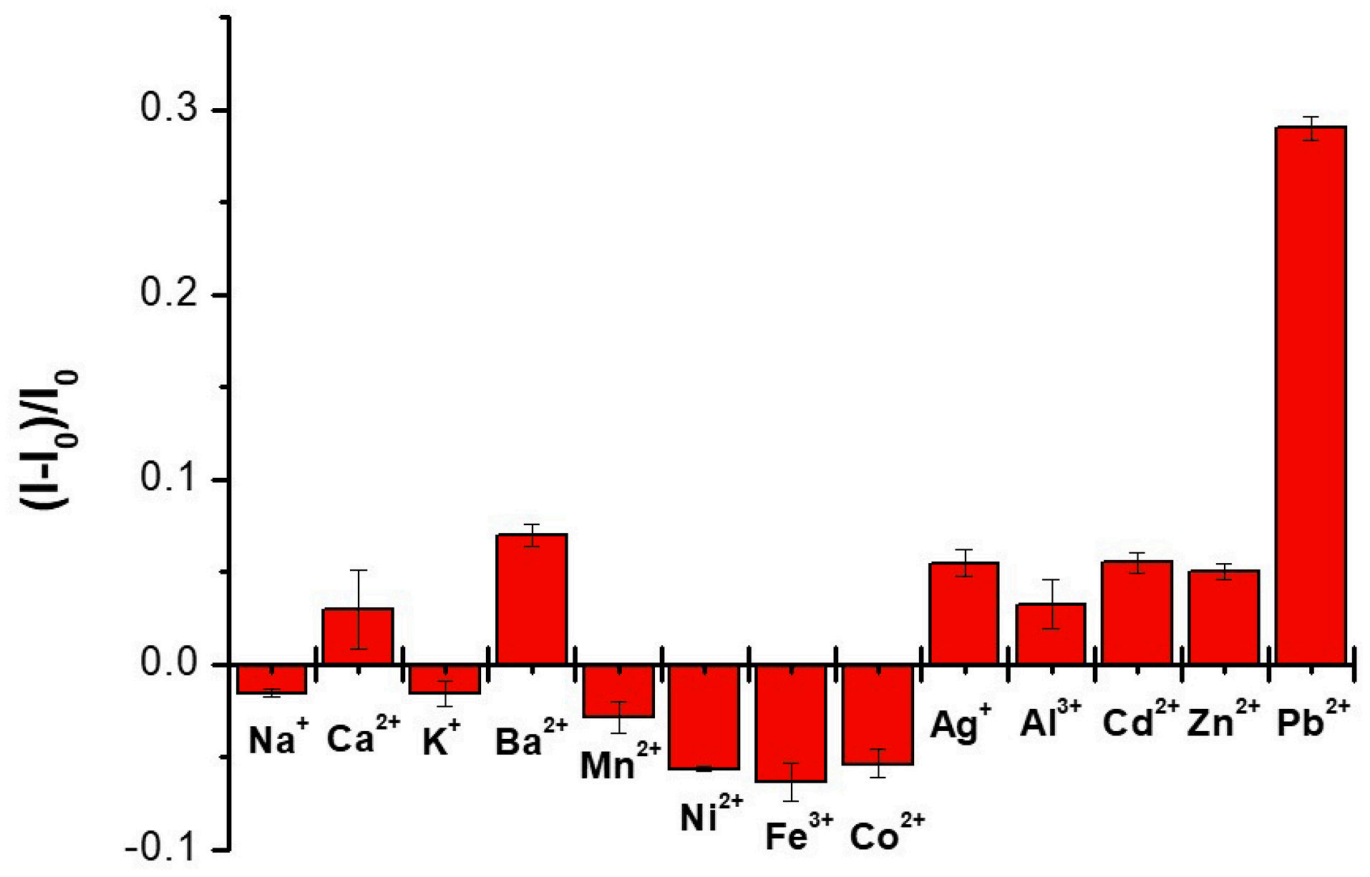
Selectivity study of the CAEA-H-PAA (mass ratio 1:12) over different metal ions. The concentration of the CAEA-H was 0.06 mg/mL at 10 mM pH 7.4 PBS buffer.

To investigate the selectivity of the sensor, the fluorescence emission spectra of CAEA-H-PAA after the addition of 1.67 mM different metal ions (Mn^2+^, K^+^, Na^+^, Ca^2+^, Pb^2+^, Zn^2+^, Ba^2+^, Fe^3+^, Co^2+^, Ni^2+^, Cd^2+^, Ag^+^, and Al^3+^) were studied. The fluorescence emission was recorded in the form of quenching ratio: (*I* – *I*_0_)/*I*_0_, where *I and I*_0_ is the fluorescence emission of CAEA-H-PAA after and before the addition of different metal ions. As is shown in Figure 8, the CAEA-H-PAA exhibits excellent selectivity of Pb^2+^ among 13 tested metal ions.

### Detection of pb2^+^ in real water samples

To study the feasibility of this C-dots-SPB probe, the spiked recovery of the CAEA-H-PAA in real water samples (Lake water, from Qiandao Lake, Zhejiang Province; natural mineral water, from Mount Huangshan City; and tap water in the laboratory from Shanghai) has been carried out via addition of different concentration of Pb^2+^ (0.667, 1, 1.33 mM). As shown in Table S2, for different water samples, the sensor achieved good recoveries of 90.6–108%. And relative standard division is < 2.13%.

### Fluorescence “turn on” mechanism for the detection of pb2^+^

Different from the traditional fluorescence quenching mechanism that is based on the electron and fluorescent resonance energy transfer process (Sun et al., 2016), the AIEE fluorescence enhancing process is based on the aggregation behavior of the carbon dots. In principle, in the aggregates, the intramolecular motion is restricted, which block the nonradiative path and activated radiative decay (Wang et al., 2016). As shown by the fluorescence emission spectra excited at 410 nm (Figure S2), the emission peaks of CAEA-H-PAA brushes slightly blue shifted to a smaller value from 491 to 470 nm, indicating the specific interaction between CAEA-H and PAA brushes (Xu et al., 2018).

In order to further understand the role that PAA brushes play in the enhancement of AIEE detection, we conducted a series of characterizations including SAXS, turbidity titration and Zeta-potential analysis.

**Figure 9:**
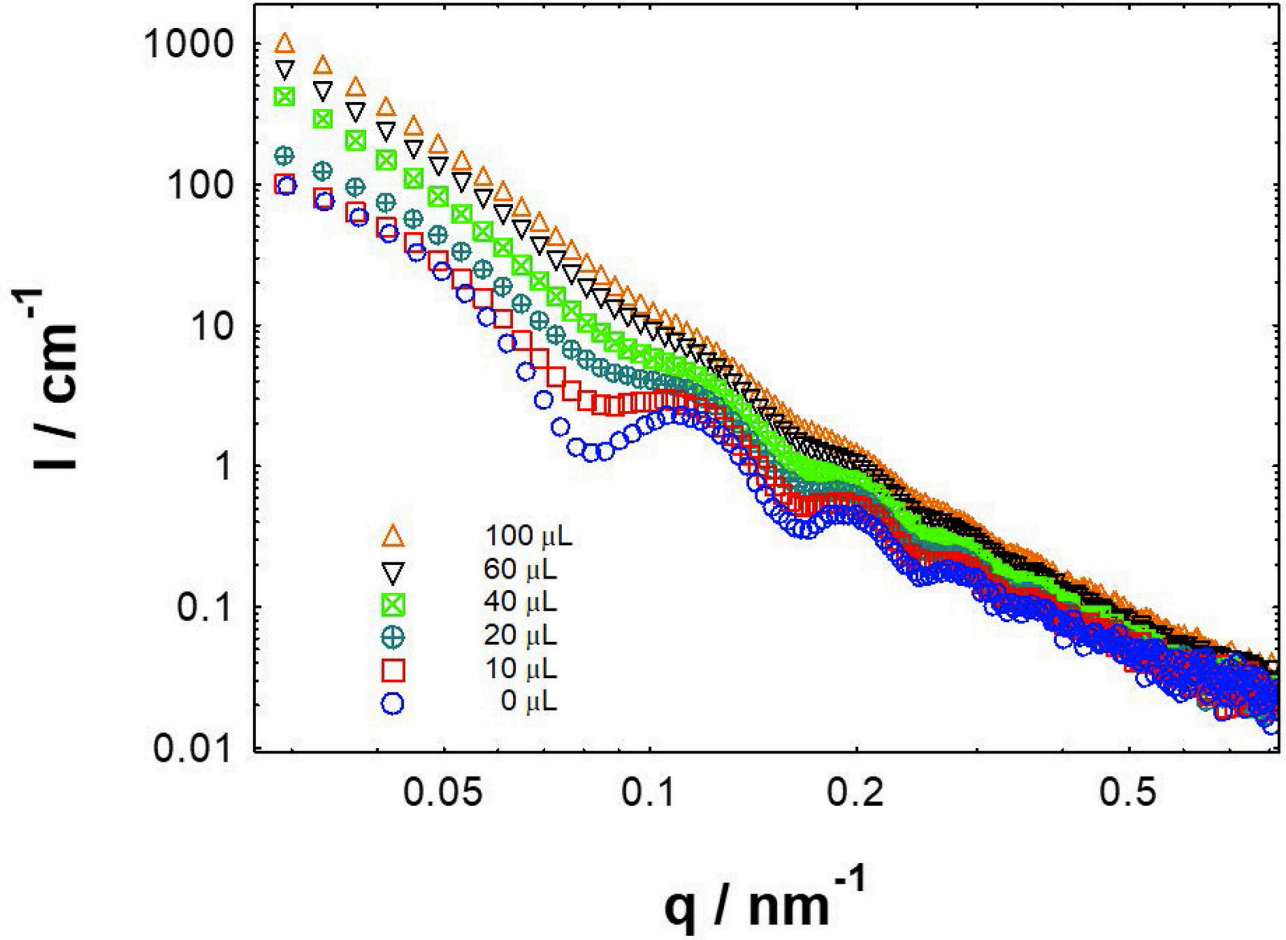
SAXS results of CAEA-H-PAA (CAEA-H / SPB mass ratio 1:12) aqueous solution at different Pb^2+^ adding amounts. CAEA-H-PAA SPB concentration was 3.9 mg/mL in pH 7.4 10 mM PBS buffer. The curves were measured by adding different volumes of 0.0167 M Pb^2+^ aqueous solution into CAEA-H-PAA solution.

Herein we conducted the SAXS tests for CAEA-H-PAA with the different amounts of Pb^2+^ and other 4 metal ions (Figures S4), respectively. As shown in Figure 9, when Pb^2+^ was added to CAEA-H-PAA solution, the scattering oscillation became gradually obscure and shift to a higher value as the Pb^2+^ concentration was increased. The obscure of the scattering oscillation from the SAXS curve is due to the increase of polydispersity which is caused by the aggregation of particles (Hilfiker et al., 1990). Moreover, the significant increase of the scattering intensity in the middle *q* region (0.1–0.3 cm^−1^) suggested the possible accumulation of CAEA-H among the PAA chains due to the aggregation of SPB particles (Tian et al., 2016).

Compared with other metal ions (Figure S3), only the scattering curves of Pb^2+^ on CAEA-PAA showed a distinct increase in polydispersity with the increase of Pb^2+^ concentration, indicating the trend of aggregation. Also, without the immobilization of CAEA-H (Figure S4), only Pb^2+^ could obscure the scattering oscillation (Figure S4) among the 5 metal ions. The SAXS data suggest the specific aggregation behavior between SPB and Pb^2+^ makes CAEA-H-SPB an ideal potential sensor to selectively detect Pb^2+^.

**Figure 10:**
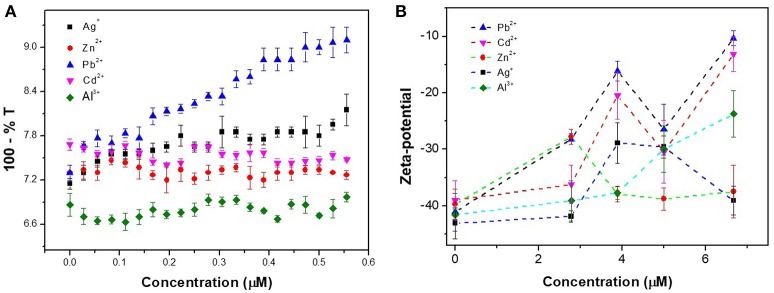
**(A)** Turbidity titration for different metal ions in CAEA-H-PAA SPB salt-free aqueous solution. The concentration of CAEA-H-PAA SPB solution was 0.013 mg / mL at pH 6.8. **(B)** Zeta-potential of CAEA-H-PAA SPB salt-free aqueous solution at different concentration of metal ions. The concentration of CAEA-H-PAA SPB solution was 0.065 mg/mL at pH = 6.8.

Other characterization methods were also used to confirm the specific aggregation behavior between CAEA-H-PAA and Pb^2+^. In Figure 10A, with the increase of Pb^2+^ concentration, the turbidity gradually increased, while the turbidity of other 4 metal ions remained stable.

As shown in Figure 10B, compared to other metal ions, the absolute value of Pb^2+^ Zeta-potential was the least for all the concentrations, indicating that the Pb^2+^-CAEA-H-PAA system was most unstable among the 5 different metal ions and was more likely to aggregate.

As is shown in Figure S5. the diameter and the polydispersity of CAEA-H-PAA after the addition of Pb^2+^ were the largest among all tested metal ions (Pb^2+^, Zn^2+^, Al^3+^, Ag^+^, Cd^2+^), further indicating the specific aggregating behavior of CAEA-H-PAA after the adding of Pb^2+^.

Additionally, the optical and fluorescence microscopy has been conducted to further confirm the specific aggregation between CAEA-H-PAA particles. As shown in the fluorescence microscopy in Figures S6A–D, only the agglomerates of CAEA-H-PAA and PAA brushes at 100 μL Pb^2+^ can be observed, which confirms the specific aggregation behavior of Pb^2+^ with CAEA-H-PAA and PAA brushes. And only CAEA-H-PAA shows a significant fluorescence emission. This suggests that the AIEE phenomenon is caused by the confining of C-dots inside SPB shell and the further aggregation of SPB particles at the presence of Pb^2+^.

Thus, by different characterization methods we could confirm the specific interaction between CAEA-H-PAA and Pb^2+^. However, it still remains to be discussed that how the aggregation behavior of SPB will enhance the fluorescence emission. To this end, 2 relevant control experiments were carried out: fluorescence titration of Pb^2+^ into CAEA-H-PAA at pH 7.4 with smaller mass ratio of SPB and CAEA-H (4:1); and fluorescence titration of Pb^2+^ into CAEA -PAA at pH 7.4 at the same mass ratio of 12:1.

As shown in Table S1, for CAEA-H-PAA at pH 7.4 with a mass ratio 4:1, only a little increase in the fluorescence emission was found (*I*/*I*_0_ = 1.02). As we have discussed in Figure 6A, when the mass ratio was 4:1 and pH = 7.4, there was no apparent electrostatic interaction between CAEA-H and SPB. In other words, there were no CAEA-H that “gathered” inside the brush shell. When Pb^2+^ ions were added to the system, further enhancement of the fluorescence emission was not significant. This proves that the CAEA-H needs to be immobilized into the brush system to enhance the fluorescence emission. Moreover, the rather weak fluorescence enhancement of Cd^2+^ for CAEA-H-PAA at mass ratio 12:1 and pH = 7.4 indicates that the aggregation of the PAA system was also inevitable for the “turn on” fluorescence detection. However, for CAEA-PAA at the same condition (mass ratio 12:1 and pH = 7.4), from Table S1 we can observe that there was no increase of the fluorescence emission either. This suggests that although the CAEA were immobilized into the brush shell, and specific aggregation of SPB happened due to Pb^2+^ addition, the fluorescence couldn't be turned on because there was no aggregation behavior between CAEA and Pb^2+^. Moreover, from previous literature (Morris et al., 1997), it has been confirmed that at basic condition, large amounts of Pb(II) would be adsorbed in the Acrylic Acid copolymer microgels, which might cause the specific aggregation between SPB particles. Thus, we can draw the conclusion now that the fluorescence “turn on” sensing of Pb^2+^ by our CAEA-H-PAA system is based on the combination of immobilizing CAEA-H into PAA brush shell, the specific aggregation of the CAEA-H-PAA, and the aggregation of CAEA-H.

Mechanistically, it should be noted that introduction of Pb(II) to SPB suspension results in agglomeration even in the absence of C-dots. For C-dot decorated SPB systems, this behavior suggests that C-dots experience a confined environment where their motion is severely restricted, an effect that gives rise to AIEE. In addition, in Figure S7, we studied the relationship between the concentration and the emission ratio between CAEA-H-PAA with 100 μL Pb^2+^ and the emission of free CAEA-H at the same condition. The observation that the fluorescence intensity of the agglomerated system (CAEA-H-PAA) far exceeds that of the initial C-dot solution, indicates that de-absorption of C-dots (due to competitive action from Pb(II)) should play only a minor role, if at all present. Regardless the exact features of the underlying mechanism it is clear that C-dots/SPB coupling can generate powerful systems with interesting sensing capabilities.

## Conclusion

In this paper, a novel fluorescence “turn on” sensor based on the immobilization of CAEA-H into PAA brushes was prepared. It could selectively detect Pb^2+^ among different metal ions with a wide detecting range (0–1.67 mM) and good linear correlation (*R*^2^ = 0.9958). For this fluorescence “turned on” probe, the fluorescence of CAEA-H was firstly quenched by immobilization into PAA brushes via electrostatic interaction induced quenching. The addition of Pb^2+^ caused the aggregation of SPB particles would specifically “turn on” the fluorescence of the Carbon dots. The results suggest that the AIEE process is based on the mutual contribution of CAEA-H immobilization, the specific aggregation of the CAEA-H-PAA, and the aggregation of CAEA-H within SPB shell. This novel SPB-based sensor can serve as a promising demonstration on the sensing of Pb^2+^ with a wide detecting range and controllable parameters, and a proof of showing that sensitive and selective chemical detection can be achieved via a C-dot/SPB synergistic platform.

## Author contributions

YT, AK, LL, FZ, YW, WW, QY, ZY and XG were involved in the experimental works. YT performed all the experiments and wrote the paper. WW and YT performed the fluorescence test and the synthesis of Carbon dots. QY and ZY provided the PAA brushes for SAXS test. AK and XG supervised on the establishment of the experiment scheme. LL instructed on the fitting of SAXS data and revised the SAXS fitting part of the manuscript. AK, FZ, YW and XG provided the revision to the whole manuscript.

### Conflict of interest statement

The authors declare that the research was conducted in the absence of any commercial or financial relationships that could be construed as a potential conflict of interest.
