# Supplementary material for: Facile Fluorescence “Turn on” Sensing of Lead Ions in Water via Carbon Nanodots Immobilized in Spherical Polyelectrolyte Brushes
Source: Front Chem. 2018 Oct 9;6:470. doi: 10.3389/fchem.2018.00470 (PMC6189401; doi:10.3389/fchem.2018.00470)
Supplement: Supplementary file 1 [file Supplementary_Material.docx]

*Supplementary Material*

Facile Fluorescence “Turn on” Sensing of Lead Ions in Water via Carbon Nanodots Immobilized in Spherical Polyelectrolyte Brushes

**Yuchuan Tian^1^, Antonios Kelarakis^2*^, Li Li^1*^,** **Fang Zhao^1^, Yunwei Wang^1^, Weihua Wang^3^, Qingsong Yang^1^, Zhishuang Ye^1^, and Xuhong Guo^1,4*^**

**Correspondence**: Prof. Antonios Kelarakis, E-mail: akelarakis@uclan.ac.uk; Porf. Li Li, E-mail: lili76131@ecust.edu.cn; or Prof. Xuhong Guo, E-mail: [guoxuhong@ecust.edu.cn](mailto:guoxuhong@ecust.edu.cn).

#### The calculation of immobilization ammounts

According to the PL data, the emission efficiency (I/I_0_) for CAEA-H before and after loading in SPB was 26%. Which means at least only 26% of the C-dots wasn’t immobilized into SPB. The immobilization amount M could be obtained from

$M=\frac{(I_{0}-I)}{I_{0}}\times\frac{C_{CDS}}{C_{SPB}}$ (1)

Where *I­*_0_ is the emission intensity of free C-dots, *I* was the emission intensity after immobilizing into SPB, *C*_CDS_ is the overall concentration of the C-dots and C_SPB_ is the concentration of the PAA brushes. As a result, the maximum immobilized amount of C-dots is calculated to 0.05 (mg/mg SPB). From SAXS results, the amount of C-dots could be approximately calculated from

$M=\frac{4}{3}\pi\sum_{i=1}^{5} \left[\Delta\rho_{i}\cdot\frac{(r_{i}^{3}-r_{i-1}^{3})}{Na}\right]\cdot\frac{\sum_{j} M_{j}}{\sum_{j} N_{j}}/M_{SPB}$ (2)

where Δ*ρ*_i_ is the difference between the excess electron distribution of SPB and CAEA-H-PAA, *Na* is Avogadro constant, *N*_j_ is the atomic number of C, N, O, H. *M*_j_ is the molar mass of C, N, O, and H. In our case, the amount of H in the CAEA-H is neglectable (< 5%). Thus the ratio between *M*_j_/*N*_j_ for C,N,O is the constant 2. *M*_SPB_ is the mass of a single SPB particle. From the calculation the immobilization amount was ca. 0.04 (mg/mg SPB).

#### DLS


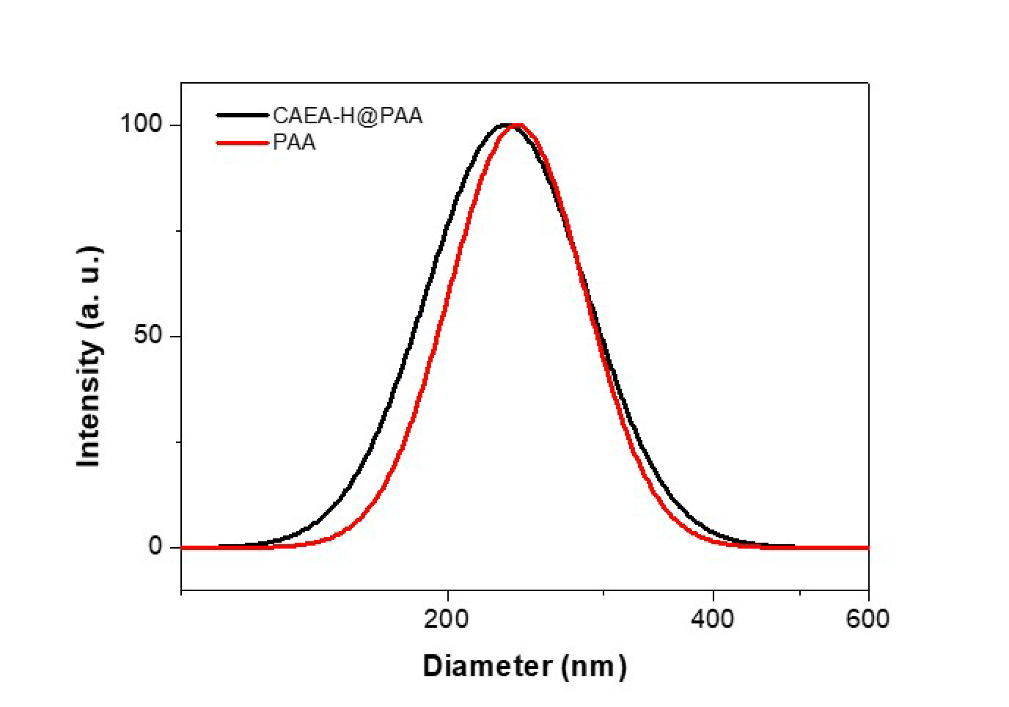


Figure S1 DLS results of PAA and CAEA-H@PAA, at 10 mM pH 7.4 PBS buffer.

#### Fluorescence emission spectra


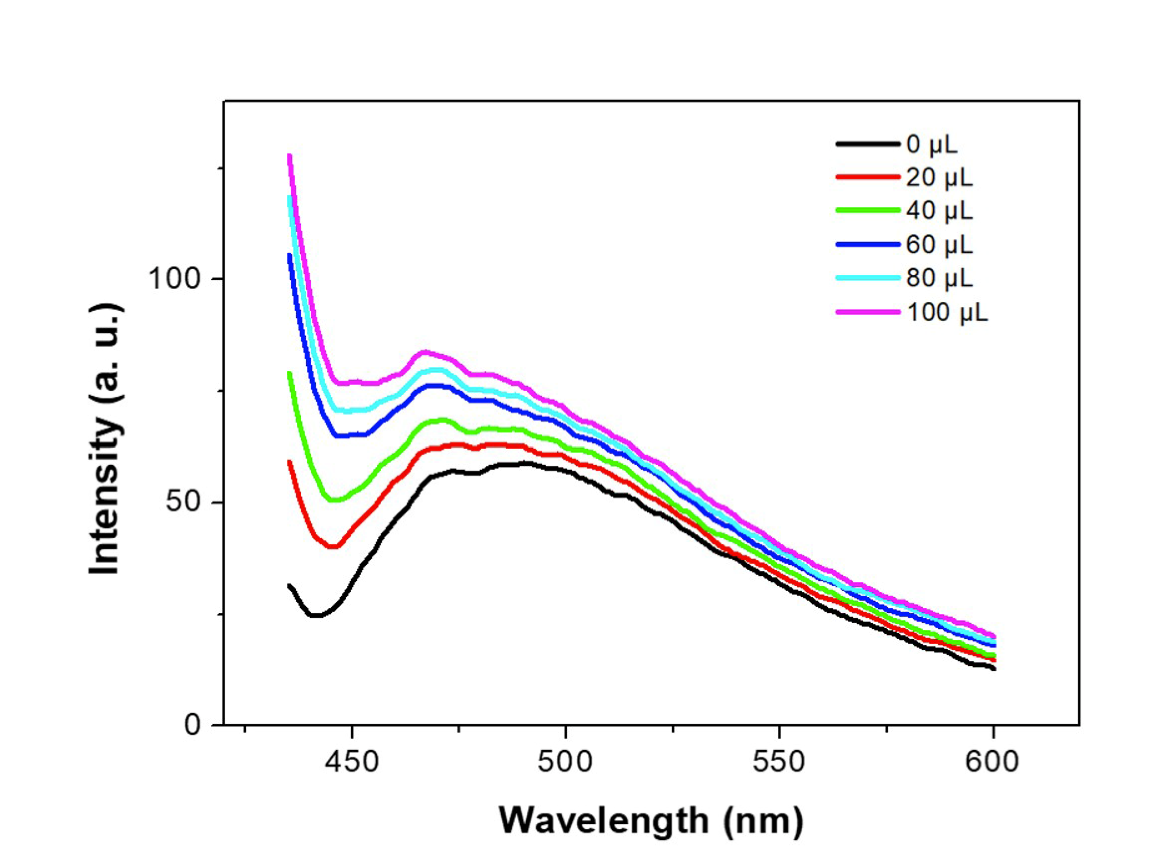


Figure S2 Fluorescence emission spectra of CAEA-H@PAA (1:12) at 10mM pH7.4 PBS solution, the mass concentration of CAEA-H is 0.06 mg / mL.

#### SAXS results


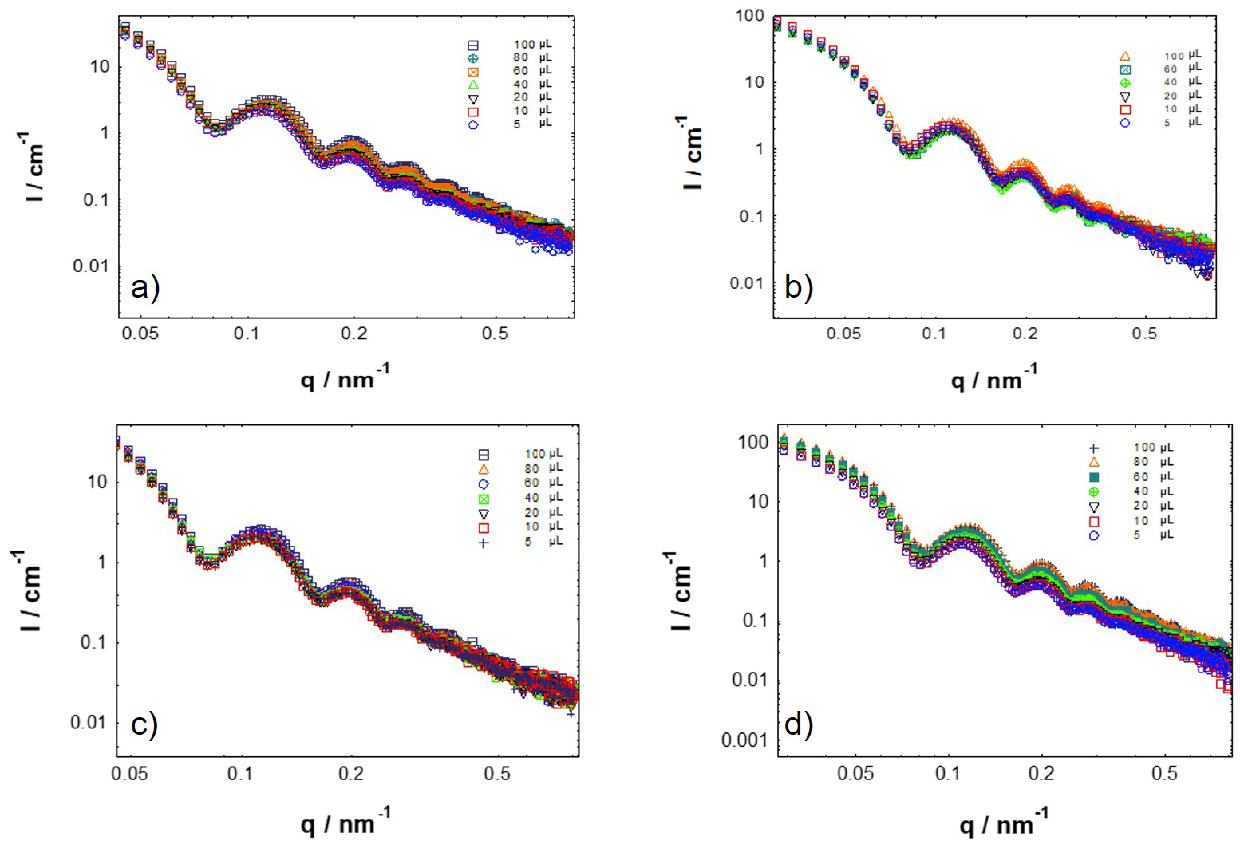


Figure S3 SAXS results of CAEA-H@PAA (CAEA-H / PAA mass ratio: 1:12) aqueous solution at different ions adding amount. CAEA@PAA SPB concentration is 3.9 mg / mL at pH 7.4 10 mM PBS buffer, the concentration of the metal ions were 0.0167M. From (a) to (d): Ag^+^, Al^3+^, Zn^2+^, Cd^2+^.


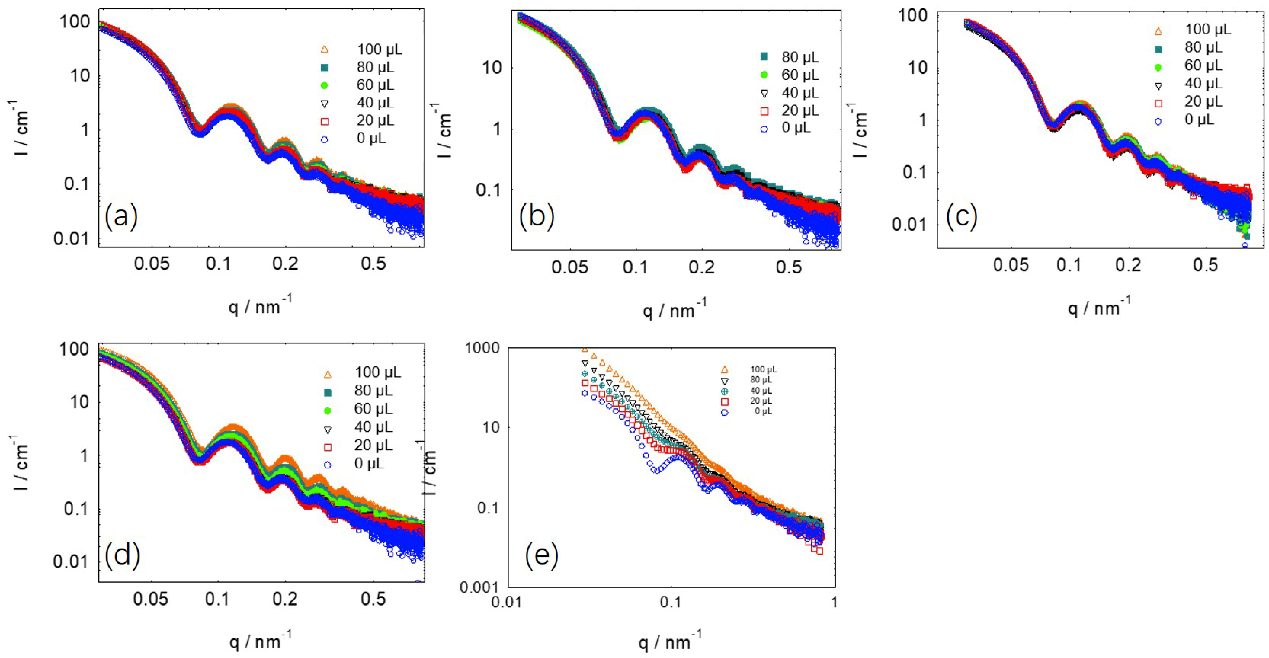


Figure S4 SAXS results of PAA aqueous solution at different ions adding amount. PAA concentration is 3.6 mg / mL at pH 7.4 10 mM PBS buffer, the concentration of the metal ions were 0.0167M. From (a) to (e): Ag^+^, Al^3+^, Zn^2+^, Cd^2+^, Pb^2+^.

#### DLS

a)b)

**Figure S5** Diameter (a) and Polydispersity (b) obtained from DLS measurement when adding different amount of metal ions into CAEA-H-PAA 12:1 at pH 7.4.

#### Optical / Fluorescence Microscopy


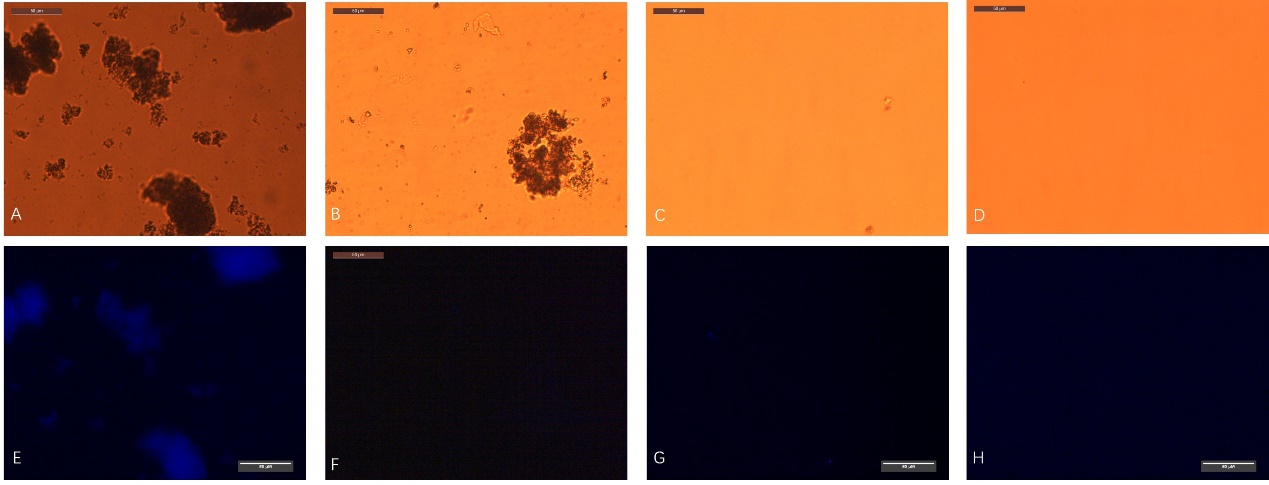


**Figure S6** Optical microscopy of different samples with the addition of 100 μL Pb^2+^: (A) CAEA-H-PAA, (B) PAA brushes, and (C) CAEA-H; Fluorescence microscopy of of different samples with the addition of 100 μL Pb^2+^: (E) CAEA-H-PAA, (F) PAA brushes, (D) CAEA-H; Optical microscopy (G) and fluorescence microscopy (H) of CAEA-H-PAA with the addition of Ca^2+^.(The concentration of CAEA-H and PAA brushes was fixed at 0.06 mg/mL and 0.72 mg/mL respectively). The excitation of fluorescence microscopy: 390 - 410 nm.

#### The influence of Concentration on I(CAEA-H-PAA) / I(CAEA-H)


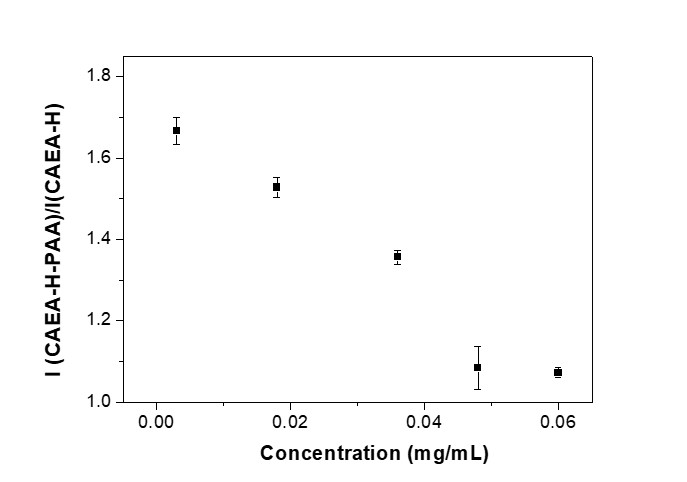


**Figure S7**. the emission ratio between CAEA-H-PAA after addition 100 μL Pb^2+^ and free CAEA-H without adding Pb^2+^ at the same condition

The experiment was carried out via changing the volume ratio between CAEA-H-PAA and phosphate buffer (10 mM pH 7.4).

#### Control experiments

Table S1 Fluorescence emission ratio (I/I_0_) of different C-dots based fluorescence sensor at different Pb concentration at 10 mM PBS buffer. The concentration of C-dots was fixed at 0.06 mg/mL.

| pH | Type | Intensity (I/I0) | 0ul | 20ul | 40ul | 60ul | 80ul | 100ul |
| --- | --- | --- | --- | --- | --- | --- | --- | --- |
| 7.4 | CAEA | Pd2+ | 1 | 0.99 | 0.99 | 0.99 | 0.99 | 0.99 |
|  | PAA : CAEA-H = 4 : 1 | Pd2+ | 1 | 1.01 | 1.03 | 1.02 | 1.01 | 1.01 |
|  | PAA : CAEA = 12 : 1 | Pd2+ | 1 | 1.01 | 1.00 | 1.00 | 0.99 | 1.00 |

#### Sensing of Pb^2+^ in real samples

Table S2 Determination of Pb^2+^ in real samples

| Sample^α^ | Added (mM) | Found (mM) | Recovery (%) | RSD (%, n = 3) |
| --- | --- | --- | --- | --- |
| Tap water | 0.667 | 0.707 | 105 | 1.42 |
|  | 1.00 | 0.906 | 90.6 | 0.278 |
|  | 1.33 | 1.43 | 107 | 1.25 |
| Mineral water | 0.667 | 0.672 | 101 | 1.89 |
|  | 1.00 | 1.02 | 102 | 0.538 |
|  | 1.33 | 1.37 | 103 | 0.535 |
| Lake water | 0.667 | 0.712 | 107 | 0.761 |
|  | 1.00 | 0.990 | 99.0 | 0.909 |
|  | 1.33 | 1.26 | 94.9 | 2.13 |

^α^ in the test, lake water was from Qiandao Lake, Zhejiang Province, mineral water was the natural underground water from Tangshu village, Huangshan City, and tap water was collected in the laboratory from Shanghai.
